# Abundance and Diversity of Bacterial, Archaeal, and Fungal Communities Along an Altitudinal Gradient in Alpine Forest Soils: What Are the Driving Factors?

**DOI:** 10.1007/s00248-016-0748-2

**Published:** 2016-03-09

**Authors:** José A. Siles, Rosa Margesin

**Affiliations:** Institute of Microbiology, University of Innsbruck, Technikerstrasse 25, A-6020 Innsbruck, Austria

**Keywords:** Soil metagenomics, Climate change, Elevational gradient, Bacteria, Archaea, Fungi

## Abstract

Shifts in soil microbial communities over altitudinal gradients and the driving factors are poorly studied. Their elucidation is indispensable to gain a comprehensive understanding of the response of ecosystems to global climate change. Here, we investigated soil archaeal, bacterial, and fungal communities at four Alpine forest sites representing a climosequence, over an altitudinal gradient from 545 to 2000 m above sea level (asl), regarding abundance and diversity by using qPCR and Illumina sequencing, respectively. Archaeal community was dominated by *Thaumarchaeota*, and no significant shifts were detected in abundance or community composition with altitude. The relative bacterial abundance increased at higher altitudes, which was related to increasing levels of soil organic matter and nutrients with altitude. Shifts in bacterial richness and diversity as well as community structure (comprised basically of *Proteobacteria*, *Acidobacteria*, *Actinobacteria*, and *Bacteroidetes*) significantly correlated with several environmental and soil chemical factors, especially soil pH. The site at the lowest altitude harbored the highest bacterial richness and diversity, although richness/diversity community properties did not show a monotonic decrease along the gradient. The relative size of fungal community also increased with altitude and its composition comprised *Ascomycota*, *Basidiomycota*, and *Zygomycota*. Changes in fungal richness/diversity and community structure were mainly governed by pH and C/N, respectively. The variation of the predominant bacterial and fungal classes over the altitudinal gradient was the result of the environmental and soil chemical factors prevailing at each site.

**Electronic supplementary material:**

The online version of this article (doi:10.1007/s00248-016-0748-2) contains supplementary material, which is available to authorized users.

## Introduction

Mountain ecosystems have attracted great interest of global ecologists over the last 200 years since these habitats are characterized by altitudinal gradients, which show dramatic changes in climate and biotic characteristics over short geographic distances. Altitudinal gradients have been used as “natural experiments” to know the effect of many environmental factors such as temperature, precipitation, atmospheric pressure, solar radiation, or clear-sky turbidity on biota [1, 2]. As a result, many studies have documented the effect of elevational patterns on a wide variety of taxonomic groups of microorganism and macroorganism [3]. In this way, it has been possible to conclude that the diversity of animals and plants follows the following three altitudinal patterns: diversity decreases monotonically with increasing altitude [4], diversity is high across the lower elevations and then decreases at middle to high altitudes, or diversity shows a hump-shaped relationship with a mid-altitudinal peak in richness [5]. However, studies on soil microbial diversity over altitudinal gradients indicate that these patterns cannot be applied for microorganisms and contradictory conclusions have been drawn from different works [6]. For example, no significant differences were found in soil bacterial diversity, using pyrosequencing, along altitudinal gradients in eastern Peru, Appalachian Mountains, and southwestern highlands of Saudi Arabia [5, 7, 8]. Meng et al. [4] found the highest bacterial diversity at medium elevations in the Mountain Lushan, China, while Singh et al. [9] found a higher bacterial diversity at higher altitudes than at medium altitudes on Mt. Halla, South Korea. Therefore, there are no obvious geographical trends in soil bacterial diversity with altitude.

Furthermore, the factors driving the variations of microbial communities over altitudinal gradients are still unknown and some studies, using even geologically uniform sites, show contradictory conclusions [10, 11]. Most of these studies are focused on bacterial communities or some of them also on archaea [1, 12], while the number of surveys studying fungal communities along altitudinal gradients and the factors modeling them is really scarce [4, 13]. Likewise, most of the aforementioned surveys are limited to description of microbial community composition, neglecting microbial abundance patterns in altitudinal gradients, but it is necessary to achieve integrative studies taking into account both microbial abundance and composition. In the same way, since archaea, bacteria, and fungi are altogether responsible for nutrient cycling, maintenance of structure in soil, and establishing symbiotic and antagonist relationships with plants [14], it is important to perform surveys considering overall prokaryotic and fungal abundances and composition in mountain systems to approach a better understanding of underlying mechanisms that determine microbial life in these ecosystems.

The Alps constitute a dominant feature of the landscape in Europe, with important economic and social implications [15]. Global warming has already increased the temperature during twentieth century by up to 2 °C in this area [16] and, under the best of the scenarios, about 0.25 °C warming per decade is expected until the mid of the twenty-first century [15]. The role of soil microorganisms in the future context of global warming is crucial since climate change involves an increase in temperature and levels of CO_2_, most likely resulting in changes in soil microbial communities’ composition, which can enhance ecosystem feedback to climate through microbial decomposition of soil organic matter (SOM), producing more greenhouse gas emissions to the atmosphere [17]. Most of current studies about the effect of climate change on soil are based on soil warming, precipitation manipulation, or increasing CO_2_ concentration [18–20]. These experiments have provided useful information at short term; however, they offer little insight into responses that occur at long term. In this context, altitudinal gradient studies help fill this gap by exploiting “space-for-time” substitution [21]. Consequently, studies on microbial abundance and diversity along altitudinal gradients in Alpine forest soils could be especially interesting since these works are helpful for a better understanding of the response of soil microorganisms to future climate change. To date, only Margesin et al. [22] and Djukic et al. [23] have studied the shifts in soil microbial community structure over elevational gradients in European Alps using colony-forming unit (CFU) counts, phospholipid fatty acid (PLFA) analysis, or fluorescence in situ hybridization (FISH). Thus, to the best of our knowledge, we here present the first study describing changes in soil archaeal, bacterial, and fungal communities from an altitudinal gradient in European Alpine forests using more accurate and informative tools, such as high-throughput sequencing techniques.

The objectives of the present study were (i) to assess abundance of all three domains (archaea, bacteria, and fungi) along an altitudinal gradient in forest soils from European Alps (using quantitative PCR (qPCR)); (ii) to describe the composition of the different microbial communities that these soils harbor (using Illumina sequencing); (iii) to analyze the diversity patterns that these communities show over the altitudinal gradient; and (iv) to determine the main environmental factors explaining the shifts in microbial abundance, diversity, and composition along the altitudinal gradient.

## Materials and Methods

### Site Description

The study sites are located in South Tyrol, in the Italian Alps. Four sites (M, K, R, and S) were selected across an altitudinal gradient from 545 to 2000 m above sea level (asl), representing a climosequence, including submontane, montane, subalpine, and alpine vegetation zones. All four sites were SW exposed and contained the same bedrock (rhyolite). The investigated sites represent widely distributed and forestally significant forest types in this part of the Italian Alps. A detailed description of each site has previously been reported by Siles et al. [24]. The main characteristics of the studied sites are summarized in Table 1.

**Table 1:** Main characteristics of the investigated sites

Characteristics	M	K	R	S
Location	Kleiner Priol (Montiggl)	Klobenstein (Ritten)	Kleebach (Ritten)	Schwarzseespitze (Ritten)
Coordinates	N 46° 25′ 36.8 ″	N 46° 32′ 38.1 ″	N 46° 35′ 16.2 ″	N 46° 35′ 21.4 ″
	E 11° 17′ 48.6 ″	E 11° 28′ 16.1 ″	E 11° 26′ 4.9 ″	E 11° 27′ 2.4 ″
Altitude (m asl)	545–570	1175–1200	1724–1737	1965–2000
Exposition	SW	SW	SW	SW
MAT (°C)	11.0	7.4	4.0	2.4
MAP (mm)	900	950	1000	1050
Altitudinal vegetation belt	Submontane	Montane	Subalpine	Alpine
Vegetation	Mixed deciduous forest	Mixed deciduous forest	Coniferous forest	Tree line
Dominant plant species	*Quercus pubescens*	*Fagus sylvatica*	*Picea abies*	*Pinus mugo*
	*Quercus robur*	*Pinus sylvestris*	*Pinus cembra*	*P. cembra*
	*Fraxinus ornus*	*P. abies*	*Larix decidua*	*P. abies*
	*P. sylvestris*	*L. decidua*	*Vaccinium myrtillus*	*Rhododendron ferrugineum*
	*Ostrya carpinifolia*			
Bedrock	Rhyolite (quartz-porphyry)	Rhyolite (quartz-porphyry)	Rhyolite (quartz-porphyry)	Rhyolite (quartz-porphyry)
Soil type	Dystric cambisol	Dystric cambisol	Haplic podsol	Haplic podsol

*asl* above sea level, *MAT* mean annual air temperature, *MAP* mean annual precipitation

### Soil Sampling

At the four sites, eight sampling stations distributed uniformly over each site (100 × 100 m) were chosen to cover within-site variability. Soil samples were collected of these sampling stations from *A*_*h*_ horizon (top 10 cm); the number of subsamples (2–5) depended on the thickness of the *A*_*h*_ horizon at each site. The distance between sampling stations in each sampling area was site-dependent. Sampling was carried out in late spring 2014 (sites M and K, 24 April 2014, and sites R and S, 3 June 2014), taking into account the different vegetation periods at the investigated sites. The 32 composite samples were transported in cooled boxes to the laboratory and immediately sieved (2-mm mesh). Subsequently, the composite samples were stored at 4 °C prior to processing for chemical analyses and at −80 °C prior to molecular analyses.

### Soil Physicochemical Characterization and Soil Temperature

Each soil sample was characterized regarding pH (CaCl_2_), content of humus (SOM), total organic carbon (TOC), total nitrogen (N), plant-available phosphorus (P), potassium (K), and magnesium (Mg), as well as electrical conductivity (EC; converted to electrolyte concentration) according to standard procedures [25–30]. C/N ratio was calculated as TOC/N. All the results were calculated on a soil dry weight basis (105 °C).

Soil temperature was measured in triplicate at each site at 4-h intervals during 1 year using DS1921G Thermochron iButton dataloggers buried at a depth of ca. 5 cm in the *A*_*h*_ horizon (DS1921G-F5#, Maxim Semiconductor Inc.).

### DNA Extraction

Total DNA from each soil sample was extracted using 250 mg of soil fresh mass using Power Soil^™^ DNA Isolation Kit (MO BIO Laboratories Inc., Solana Beach, USA) following the manufacturer’s instructions. Subsequently, all DNAs were quantified using QuantiFluor^™^ dsDNA System (Promega, Madison, USA) and DNA concentration for each extraction was standardized to 20 ng μL^−1^. Finally, the original eight extracts of nucleic acids from each site were merged two by two to give four pooled pair of composite samples.

### qPCR

The relative abundances of archaeal, bacterial, and fungal communities at the studied sites were assessed for each DNA extract by qPCR using a Corbett Life Science (Qiagen, Valencia, USA) Rotor-Gene 6000 system and SYBR^®^ Green as detection system (Bio-Rad, Hercules, USA). For prokaryotic communities, a fragment of the 16S ribosomal ribonucleic acid (rRNA) gene was amplified using the pairs of primers Arch-967F/Arch-1060R [31] for archaea and Eub338/Eub518 for bacteria [32]. For fungi, a fragment of 18S rRNA gene was amplified using FR1/FF390 primers [33]. The domain specificity of each pair of primers was confirmed using the Ribosomal Database Project (http://rdp.cme.msu.edu/). Each 20 μL reaction contained 10 μL iQ^™^ SYBR® Green Supermix (Bio-Rad, Hercules, USA), 0.4 μL per primer (10 μM) (VBC Biotech, Vienna, Austria), 0.4 μL bovine serum albumin (10 mg mL^−1^; New England Biolabs, Hitchin, UK), 2 μL template DNA (2 ng), and 6.8 μL H_2_O. All the samples were analyzed in duplicate on PCR strip tubes (Axygen, Thermo Fisher Scientific Inc., Waltham, USA) with the following amplification conditions: 95 °C for 15 min followed by 40 cycles of 94 °C for 60 s, 59 °C (archaea)/53 °C (bacteria)/58 °C (fungi) for 30 s, and 72 °C for 60 s. After amplification reactions, melting curve and gel electrophoresis analyses were performed to confirm that the amplified products had the appropriate size. The qPCR efficiencies were 93 % (*R*^2^ = 0.998) for archaea, 95 % (*R*^2^ = 0.999) for bacteria, and 91 % (*R*^2^ = 0.998) for fungi.

Standards for the qPCR assays were generated by PCR, amplifying each gene of interest from the genomic DNA of cultures *Methanococcus voltae* (DSM 4254) for archaea, *Sphingomonas alpine* (DSM 22537) for bacteria, and *Rhodotorula glacialis* (DSM 18768) for fungi, using the primers previously described for each kingdom. The PCR products were confirmed on an agarose gel and then cloned into a pGEM^®^-T Easy Vector System (Promega, Madison, USA), following the manufacturer’s instructions. Positive clones were isolated and extracted for plasmid DNA using a ChargeSwitch^®^-Pro Plasmid MiniPrep Kit (Invitrogen, Madison, USA). Serial dilutions of each plasmid DNA were prepared, and archaeal, bacterial, and fungal gene copy numbers were calculated using a regression equation for each assay relating the cycle threshold (Ct) value to the known number of copies in the standards as described [34]. The copy numbers of archaeal and bacterial 16S rRNA as well as fungal 18S rRNA genes were expressed as copy number per gram dry weight soil. The relations F/B (fungi/bacteria) and A/B (archaea/bacteria) were calculated for each site on the basis of the number of copies of 16S rRNA or 18S rRNA genes log transformed.

### 16S rRNA Gene Fragment and Internal Transcribed Spacer 1 Sequencing

Archaeal and bacterial communities were characterized, amplifying a fragment of 16S rRNA gene capturing V4–V5 region using the primers 515F and 806R [35]. This primer set has shown to be universal for nearly all bacterial and archaeal taxa and accurately represents taxonomic classification of sequences [36]. From each site, four DNA samples were amplified in triplicate using the HotStarTaq Plus Master Mix Kit (Qiagen, Valencia, USA) containing bar-coded forward primers, under the following thermal conditions: initial denaturation at 94 °C for 3 min, followed by 28 cycles of denaturation at 94 °C for 30 s, primer annealing at 53 °C for 40 s, and extension at 72 °C for 60 s as well as a final elongation step at 72 °C for 5 min. After amplification, reactions of the same sample were merged and the success of the amplification as well as the relative intensity of bands were checked in 2 % agarose gel. Subsequently, PCR products were purified using calibrated AMPure XP beads (Beckman Coulter, Inc., Pasadena, USA) and combined in equimolar ratios. The pooled and purified product was then used to prepare DNA library following Illumina Truseq DNA library preparation protocol. Paired-end sequencing (2 × 300) was performed on the Illumina MiSeq sequencing platform (Illumina, San Diego, USA) at MR DNA (www.mrdnalab.com; Shallowater, TX, USA).

Fungal communities were analyzed by amplification of internal transcribed spacer 1 (ITS1) region using the ITS1F and ITS2 primers [37], using the following cycling conditions: 94 °C for 3 min, followed by 28 cycles of 94 °C for 30 s, 57 °C for 40 s, and 72 °C for 60 s, after which a final elongation step at 72 °C for 5 min was performed. The processing of PCR products for paired-end sequencing (2 × 300) on Illumina MiSeq sequencing platform was as archaea and bacteria.

### Bioinformatic Analysis

First, raw Illumina MiSeq paired-end reads were assembled using MR DNA pipeline for 16S rRNA prokaryotic and ITS1 region fungal libraries. Subsequently, sequences were demultiplexed and formatted for processing using a Phython script (http://drive5.com/usearch/manual/uparse_pipeline.html). Next, prokaryotic and fungal sequences were separately quality-filtered and clustered into operational taxonomic units (OTUs) using UPARSE pipeline and UPARSE algorithm [38]. Briefly, sequences were quality-filtered allowing a maximum e-value of 0.5. Subsequently, reads were trimmed to 240-bp (base pairs) length as well as dereplicated and sorted by abundance, removing singletons (sequences which appeared once) prior OTU determination at 97 % sequence identity. Then, chimeric sequences were detected and removed using UCHIME [39] and Gold database as reference. Finally, reads from the entire dataset were mapped back to the representative prokaryotic or fungal databases to generate one OTU table for bacteria and archaea and another one for fungi. The taxonomic affiliation of each OTU was obtained using Ribosomal Database Project taxonomic classifier [40] against 16S rRNA training set 10 for prokaryotic sequences and UNITE Fungal ITS train set 07-04-2014 for fungal sequences using a 50 % threshold. Next, the prokaryotic OTU table was divided into two different datasets, one of them containing the OTUs classified as archaea domain and their abundances and another one containing the OTUs belonging to bacteria. These datasets were separately used for the downstream analyses. In the case of archaea, since the number of OTUs and sequences to characterize this community was extremely low, we decided to use these data only for the description of community composition.

### Diversity and Statistical Analyses

Except for rarefaction curves, the number of sequences per sample was normalized based on the number of sequences obtained from the smallest library for each community before analysis. The bacterial and fungal communities were characterized in terms of diversity for each site by calculating richness (number of OTUs), Shannon index, evenness, and the richness estimator indices Chao1 and ACE (abundance-based coverage estimation) using Mothur v.1.34.4 [41]. This program was also used to assess the differences in the structure of bacterial and fungal communities at the four sites through a non-metric multidimensional scaling (NMDS) analysis based on Bray-Curtis similarities at OTU level, and the significance of the observed differences were determined by PERMANOVA (distance-based permutational multivariable analysis of variance) using 9999 permutations. The shifts in the relative abundance of the top 15 bacterial classes and top 10 fungal classes were displayed by a heat map, which was modeled with “pheatmap” package in R [42].

Statistical differences between the sites in mean soil temperature and chemical soil properties; archaeal, bacterial, and fungal copy numbers; ratios F/B and A/B; richness, Shannon index, evenness, and richness estimator indices; and the relative abundance of the top 15 bacterial classes and top 10 fungal classes were analyzed by ANOVA, and Tukey’s honest significance difference (HSD) test was used for multiple comparison of means at a 95 % confidence interval.

Multiple-correlation analyses using Pearson’s method were used to relate archaeal, bacterial, and fungal abundances; the different diversity characteristics of bacterial and fungal communities; and the bacterial top 15 and fungal top 10 most abundant classes with environmental [altitude, mean annual air temperature (MAT), mean annual soil temperature (MAST), and mean annual precipitation (MAP)] and soil chemical factors analyzed (pH, EC, humus, TOC, N, C/N, P, K, and Mg). Although altitude is not an environmental variable itself, we used this parameter in correlation analyses since it is related to a range of variables that affect the ecosystem, such as temperature, precipitation, and vegetation richness [43]. For ANOVA and Pearson’s correlation analyses, the normality of data was confirmed by Kolmogorov–Smirnov test. Mantel test was used to study the relationship between bacterial and fungal community similarities and environmental and soil chemical factors. Bray-Curtis similarities between bacterial or fungal communities of the different sites were related to a Bray-Curtis matrix including all the aforementioned environmental and chemical factors. Mantel tests using the same metrics methods were also utilized to relate the overall environmental and soil chemical variables with microbial abundance and diversity properties. The correlations were performed using PAST version 3.06. Correlations were considered as significant when *p* ≤ 0.05 using 9999 permutations.

### Data Accessibility

The sequence data were deposited in the MG-RAST public database (http://metagenomics.anl.gov/) under accession number 4684007.3 for archaeal and bacterial sequences and 4684008.3 for fungal sequences.

## Results

### Soil Physicochemical and Temperature Properties

All four sites were carbonate-free and acidic soils; the lowest pH value (3.4) was measured at site R (Table 2). In general, higher levels of EC and higher amounts of SOM and total N were found at higher altitudes (sites R and S). There was a significantly positive relation between altitude and EC (*r* = 0.741, *p* < 0.001), altitude and humus, and TOC (*r* = 0.605, *p* < 0.001), and between altitude and total N (*r* = 0.699, *p* < 0.001). However, there was not a significant correlation between altitude and C/N (*r* = 0.236, *p* > 0.05); the site with the highest C/N ratio was K. Altogether, there was a trend of an increase in nutrients (P, K, and Mg) with altitude (Table 2).

**Table 2:** Soil physicochemical properties and mean annual soil temperature (MAST) at the sites M, K, R, and S

Factor	M	K	R	S
	(545–570 m)	(1175–1200 m)	(1724–1737 m)	(1965–2000 m)
pH	4.54 c	4.10 b	3.39 a	4.13 b
EC (mg KCl kg(dw)^−1 ^soil)	50.67 a	184.15 ab	363.93 bc	447.96 c
Humus (%)	16.61 a	39.79 ab	43.56 b	52.43 b
TOC (%)	9.67 a	23.13 ab	25.33 b	30.48 b
N (%)	0.48 a	0.75 ab	1.10 bc	1.19 c
C/N	19.94 a	29.30 c	22.58 ab	25.11 bc
P (mg kg(dw)^−1^ soil)	22.38 a	38.75 ab	43.50 ab	57.00 b
K (mg kg(dw)^−1^ soil)	120 a	370 b	347 ab	416 b
Mg (mg kg(dw)^−1^ soil)	201 a	282 b	208 ab	380 b
MAST (°C)	9.77 b	9.36 b	4.08 a	3.78 a

For each variable, data followed by different letters are significantly different according to Tukey’s HSD test (*p* ≤ 0.05)

*EC* electrical conductivity, *TOC* total organic carbon, *MAST* mean annual soil temperature

Continuous monitoring of soil temperature over 1 year showed that MAST decreased with altitude. MAST was significantly lower at the subalpine and alpine sites (R and S) than at the submontane and montane sites (M and K; Table 2).

### Archaeal, Bacterial, and Fungal Abundances

**Fig. 1:**
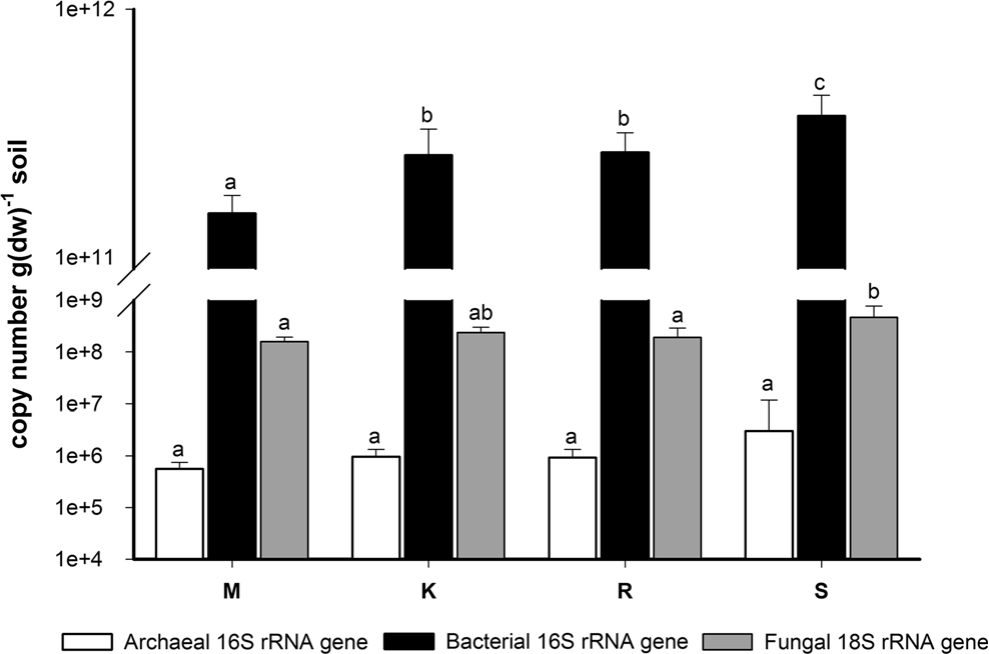
Relative archaeal, bacterial, and fungal abundances determined by qPCR at the sites M (545–570 m asl), K (1175–1200 m), R (1724–1737 m), and S (1965–2000 m). For each community, data with *different letters* are significantly different according to Tukey’s HSD test (*p* ≤ 0.05). *Bars* represent standard deviation

The archaeal 16S rRNA gene copy number did not significantly (*p* > 0.05) vary between the four sites (Fig. 1) and did not correlate with any of the environmental or chemical properties tested (Table 3). In contrast, the bacterial abundance was significantly different (*p* < 0.001) at the four sites; the 16S rRNA gene copy number increased with altitude (S > R > K > M; Fig. 1). The bacterial abundance was significantly correlated with all the environmental and chemical variables measured except pH, whereby the highest positive correlation was found with EC (Table 3). Also, the fungal abundance varied with altitude; in this case, the site with the highest copy number of 18S rRNA gene was the alpine site S (Fig. 1). All the environmental and chemical variables tested were significantly and positively correlated with fungal abundance except MAST, pH, and C/N. Mg was the factor showing the highest (positive) correlation with fungal abundance (Table 3).

**Table 3:** Mantel test and multiple-correlation analysis results considering archaeal, bacterial, and fungal abundances as well as the different environmental and chemical soil factors analyzed

Factor	Archaeal abundance	Bacterial abundance	Fungal abundance
Overall	0.216	0.710**	0.185*
Altitude	0.290	0.763**	0.433*
MAT	−0.293	−0.761**	−0.435*
MAST	0.267	−0.638**	−0.351
MAP	0.337	0.735**	0.499*
pH	0.182	−0.244	−0.028
EC	0.244	0.831**	0.584**
Humus	−0.020	0.784**	0.683**
TOC	−0.020	0.785**	0.683**
N	0.016	0.800**	0.642**
C/N	−0.059	0.438**	0.363
P	0.093	0.821**	0.752**
K	−0.073	0.772**	0.595**
Mg	0.020	0.795**	0.864**

Values in bold indicate statistical significance. Significance levels are shown at *p < 0.05 and **p < 0.01

*Overall* sum of all the factors, *MAT* mean annual air temperature, *MAST* mean annual soil temperature, *MAP* mean annual precipitation, *EC* electrical conductivity, *TOC* total organic carbon

The F/B and A/B ratios (based on log copy number) were not significantly different at the four sites (*p* > 0.05). The values for F/B varied between 0.72 ± 0.02 (mean ± SD; site R) and 0.74 ± 0.03 (site S), while the values for A/B ranged from 0.51 ± 0.02 (site M) to 0.54 ± 0.02 (site S).

### Archaeal, Bacterial, and Fungal Taxonomic Characteristics

A total of 761,110 high-quality prokaryotic sequences were obtained across the 16 samples (4 biological replicates per site) of the 4 forest sites, with an average number of 47,569 (SD = 16,985) sequences per site. These sequences were distributed between 5437 different OTUs at 97 % identity. Twenty OTUs (1466 sequences) were classified as archaea and 5401 as bacteria (759,185 sequences), and 16 (459 sequences) were unclassified using a 50 % threshold.

**Fig. 2:**
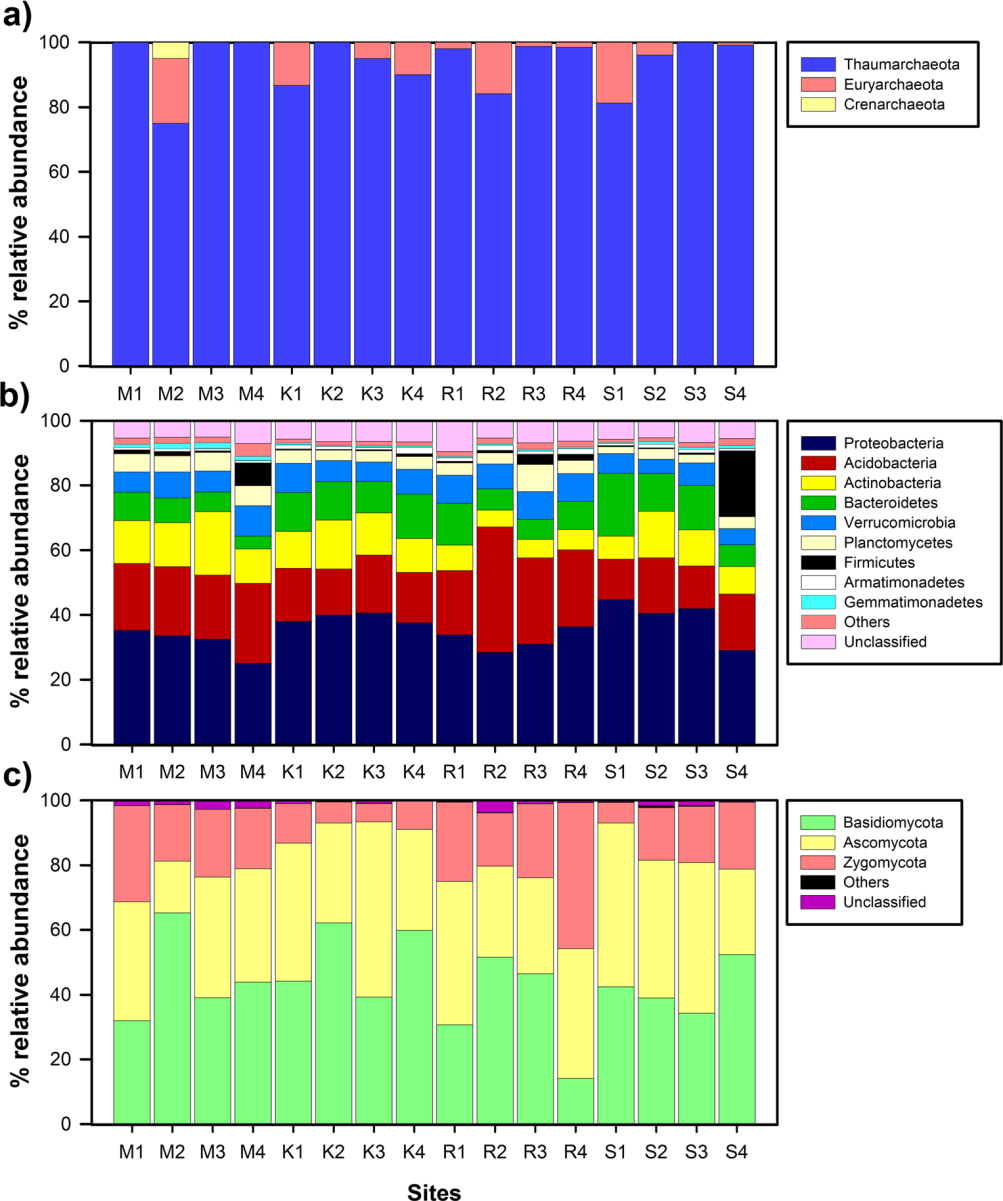
Relative abundance of the different archaeal (**a**), bacterial (**b**), and fungal (**c**) phyla found at the sites M (M1–M4; 545–570 m asl), K (K1–K4; 1175–1200 m), R (R1–R4; 1724–1737 m), and S (S1–S4; 1965–2000 m)

All the archaeal sequences could be classified at phylum level. *Thaumarchaeota* clearly dominated the archaeal community in all the samples, and no significant differences were found in the relative abundance of this phylum between the four sites (ANOVA, *p* > 0.05; Fig. 2a). All the *Thaumarchaeota* sequences belonged to *Thaumarchaeota* class and *Nitrososphaera* genus. Some sequences belonged to *Euryarchaeota* and *Crenarchaeota* phyla, although a more detailed identification of these sequences was not possible.

Regarding bacterial community, the rarefaction curves showed that the sequencing work was relatively comprehensive to cover bacterial diversity as the rarefaction curves tended to approach saturation plateau (Fig. S1). Approximately 94 % of the sequences were classified in 21 different phyla across all the samples. The predominant phyla were *Proteobacteria* (the number of classified sequences in this phylum ranged from 25.0 to 44.7 % in all the samples), *Acidobacteria* (12.6–38.8 %), *Actinobacteria* (5.2–19.6 %), *Bacteroidetes* (3.9–19.3 %), *Verrucomicrobia* (4.5–9.4 %), and *Planctomycetes* (2.1–8.4 %; Fig. 2b). The sum of sequences belonging to these phyla accounted for more than 75 % of the bacterial sequences obtained from all the samples, except for S4. At the class level, about 82 % of sequences were classified, whereby the most abundant classes were *Alphaproteobacteria*, *Gammaproteobacteria*, and *Betaproteobacteria* among *Proteobacteria*; Gp1, Gp3, and Gp2 subgroups among *Acidobacteria*; *Actinobacteria* (with *Actinomycetales* as the most common order in this class); *Sphingobacteriia* among *Bacteroidetes*; and *Planctomycetia* among *Planctomycetes* (Fig. S2a).

In the case of fungal community, Illumina analysis yielded 565,414 valid sequences across the 16 samples, with an average number of 35,338 (SD = 6648) sequences per site. The total number of sequences represented 1528 OTUs at 97 % threshold. The rarefaction curves for the analyzed samples tended to be asymptotic, meaning that fungal community was relatively deeply characterized (Fig. S3). Almost 98 % of sequences were classified in five different phyla, predominantly, *Basidiomycota* (the number of classified sequences in this phylum ranged from 14.1 to 65.3 %), *Ascomycota* (16.0–54.0 %), and *Zygomycota* (5.6–45.1 %; Fig. 2c). The other fungal phyla were *Glomeromycota* and *Chytridiomycota*. At the class level, it was possible to classify about 90 % of sequences; the most common classes were *Leotiomycetes*, *Dothideomycetes*, *Eurotiomycetes*, and *Sordariomycetes* among Ascomycota; *Agaricomycetes* and *Tremellomycetes* among *Basidiomycota*; and *Incertae sedis* 10 (*Mucorales and Mortierellales* orders) among *Zygomycota* (Fig. S2b).

### Shifts in Bacterial Diversity and Community Structure Along the Altitudinal Gradient

Bacterial richness, Shannon index, evenness, and the richness estimators Chao1 and ACE were significantly (*p* < 0.01) higher at the lowest altitude site (M) than at the other three sites, which were not significantly different (Table 4). Mantel test showed that bacterial community diversity characteristics were overall influenced by environmental factors and soil characteristics (Table S1). Soil pH showed the highest correlation with bacterial diversity indices except ACE (Table S1).

**Table 4:** Diversity characteristics of bacterial and fungal communities determined at the sites M, K, R, and S

Bacterial community				
Properties	M	K	R	S
	(545–570 m)	(1,175–1,200 m)	(1,724–1,737 m)	(1,965–2,000 m)
Number of sequences	63,309	45,234	34,270	46,982
Richness	2,199 b	1,679 a	1,425 a	1,701 a
Shannon index	6.73 b	6.19 a	5.85 a	6.13 a
Evenness	0.88 b	0.83 ab	0.81 a	0.83 a
Chao1	3,312 b	2,601 a	2,301 a	2,551 a
ACE	3,616 b	2,809 a	2,810 a	2,841 a
Fungal community				
Properties	M	K	R	S
	(545–570 m)	(1,175–1,200 m)	(1,724–1,737 m)	(1,965–2,000 m)
Number of sequences	28,835	34,408	33,495	44,614
Richness	431 b	334 a	307 a	433 b
Shannon index	3.92 b	3.23 ab	3.13 a	3.69 ab
Evenness	0.62 a	0.56 a	0.55 a	0.61 a
Chao1	516 ab	460 ab	410 a	549 b
ACE	519 ab	465 ab	413 a	544 b

For each variable and microbial community, data followed by different letters are significantly different according to Tukey’s HSD test (*p* ≤ 0.05)

**Fig. 3:**
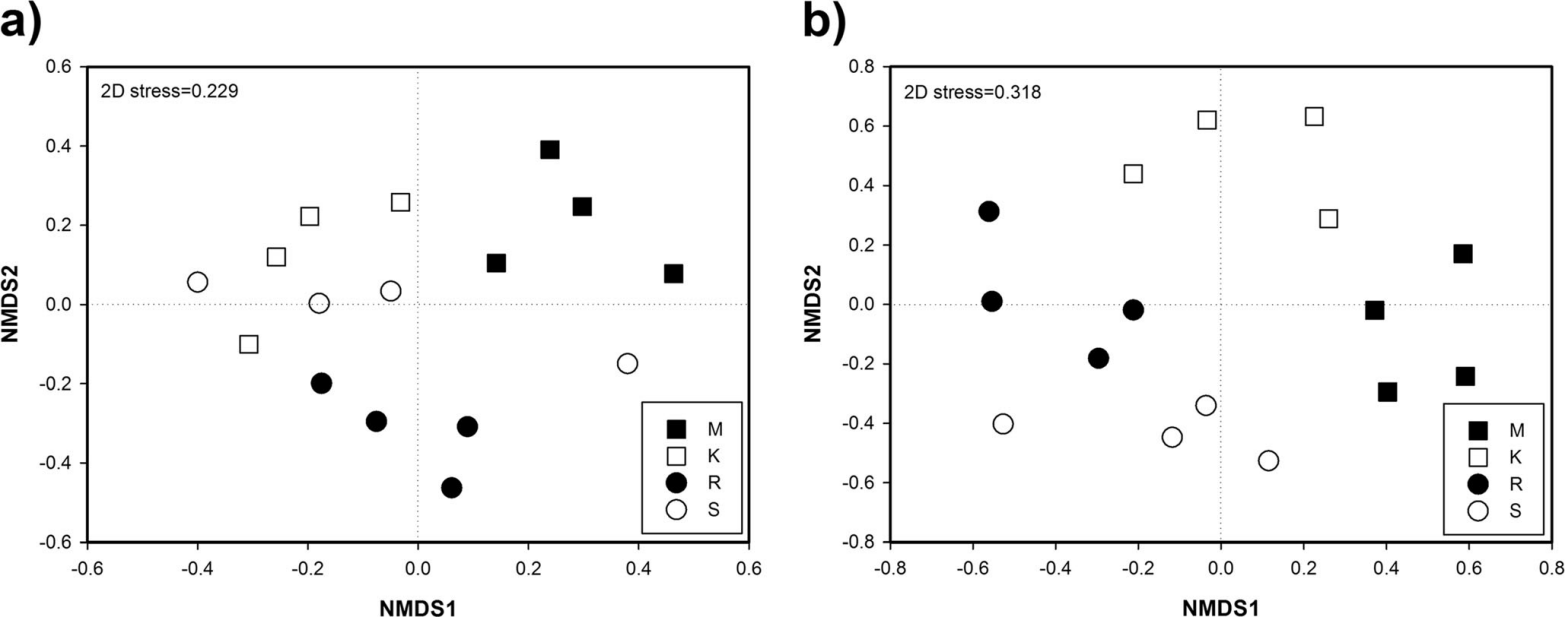
NMDS ordination based on Bray-Curtis similarities of bacterial (**a**) and fungal (**b**) communities found at the sites M (545–570 m asl), K (1175–1200 m), R (1724–1737 m), and S (1965–2000 m)

Bacterial community NMDS based on Bray-Curtis similarities showed that the most important factor determining sample ordination was the site, with similar bacterial communities at sites K and S (Fig. 3a). The significance of this clustering was tested using PERMANOVA; global PERMANOVA analysis determined that bacterial community structures at the four sites were significantly different (*p* < 0.001). However, pairwise PERMANOVA demonstrated that each pair of sites was significantly different except in the case of sites K and S (*p* = 0.855).

According to Mantel test, the bacterial community structure along the altitude gradient was influenced by all the environmental and chemical soil properties analyzed (Table 5). The Mantel test correlating each variable with bacterial community structure showed that the most important variable explaining community composition was soil pH, although other factors such as nutrient concentration (TOC, K, and Mg), EC, and humus were also important (Table 5).

**Table 5:** Mantel test results considering the bacterial and fungal community structures as well as the different environmental and chemical soil factors analyzed

Factor	Bacterial community	Fungal community
Overall	0.436**	0.510**
Altitude	0.349**	0.462**
MAT	0.236*	0.451**
MAST	0.175*	0.455**
MAP	0.257*	0.429**
pH	0.624**	0.259*
EC	0.390*	0.407**
Humus	0.372**	0.317**
TOC	0.371**	0.317**
N	0.227	0.257*
C/N	0.201	0.539**
P	0.264*	0.265*
K	0.417**	0.283**
Mg	0.461**	0.313**

Values in bold indicate statistical significance. Significance levels are shown at *p < 0.05 and **p < 0.01

*Overall* sum of all the factors, *MAT* mean annual air temperature, *MAST* mean annual soil temperature, *MAP* mean annual precipitation, *EC* electrical conductivity, *TOC* total organic carbon

**Fig. 4:**
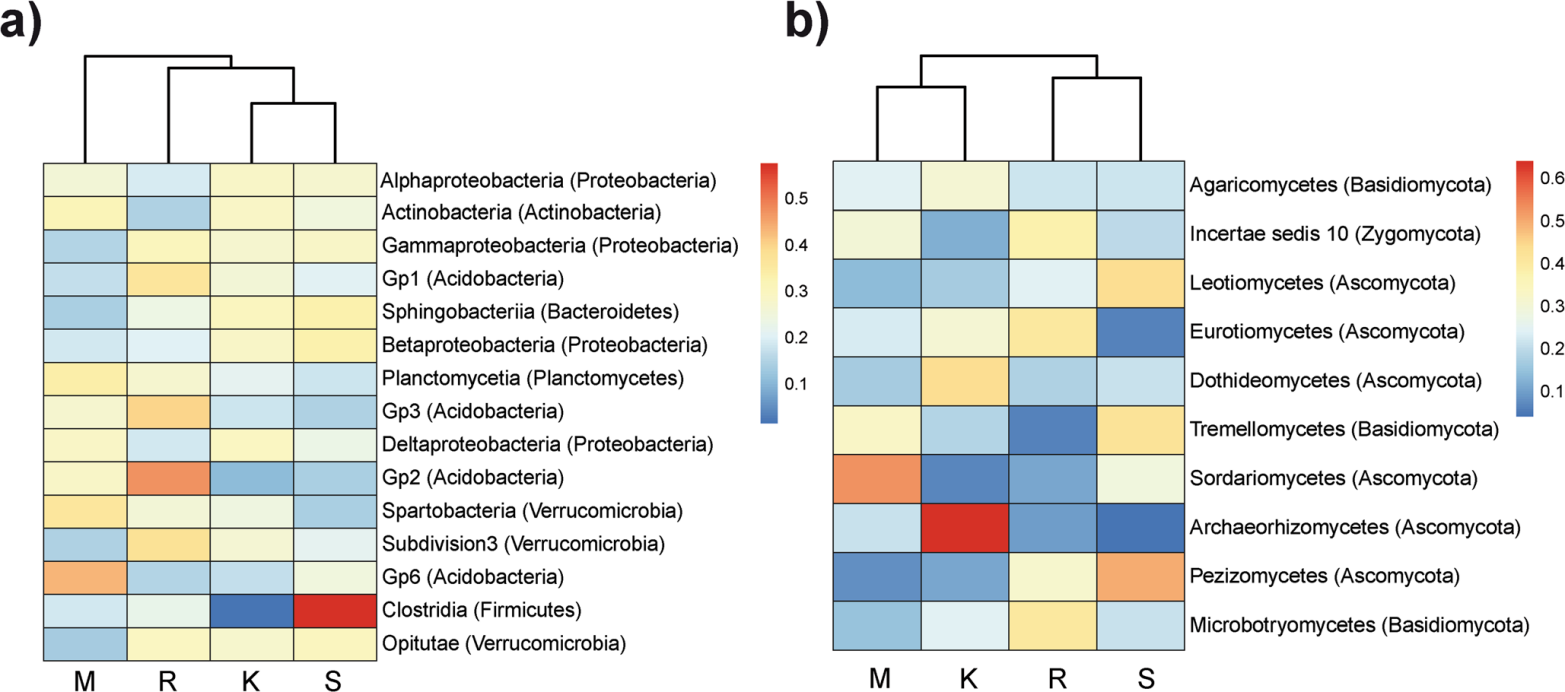
Heat map showing the relative abundance of the top 15 most abundant bacterial classes (**a**) and top 10 most abundant fungal classes (**b**) found at the sites M (545–570 m asl), K (1175–1200 m), R (1724–1737 m), and S (1965–2000 m). Sites were clustered using UPGMA dendrogram based on Bray-Curtis similarities. Color legend and scale are provided in the figure (*blue colors* mean lower relative abundance while *yellow and red colors* mean higher relative abundance)

The heat map graphically showed that the relative abundance of the top 15 most abundant bacterial classes (representing about 82 % of the total number of normalized and classified sequences) varied between the four forest sites and also supported the bacterial NMDS and PERMANOVA analyses since the UPGMA dendrogram clustered K and S sites (Fig. 4a). Among the proteobacterial classes, *Alphaproteobacteria*, *Gammaproteobacteria*, and *Betaproteobacteria* significantly varied over the altitudinal gradient (Table S2). *Alphaproteobacteria* were less abundant at site R, and the only factor significantly modeling this bacterial group was soil pH (Table S3). *Gammaproteobacteria* were present at a lower relative abundance at site M compared to the other sites and were significantly correlated with all the environmental and chemical properties tested (Table S3). The relative abundance of *Betaproteobacteria* was significantly higher at site S than at the other sites and was significantly correlated with the chemical soil factors related to nutrient concentration.

*Actinobacteria* also varied with altitude, and the correlation between altitude and *actinobacterial* abundance was significantly negative. This group was also modeled by soil pH, EC, and air and soil temperature (MAT and MAST; Table S3). Regarding *Acidobacteria*, Gp1, Gp3, and Gp2 classes were significantly more abundant at site R than at the other sites; however, Gp6 was more abundant at site M. The correlation analyses showed that all the *acidobacterial* classes were highly influenced by soil pH except Gp2, which was correlated to C/N and nutrient concentrations. Likewise, also Gp6 was related with humus, TOC, N, C/N, and K. In the case of *Sphingobacteriia* (*Bacteroidetes* phylum), it was possible to find a significantly positive correlation between its relative abundance and altitude, although the highest *sphingobacterial* abundance was found at site K. Many other factors also correlated with this bacterial class, except soil pH and MAST (Table S3). *Planctomycetia* class (*Planctomycetes* phylum) distribution was significantly influenced by most of the factors tested, especially the amount of TOC and humus (contrary to *Sphingobacteriia*). There was a negative correlation between the abundance of this class and the altitude.

### Shifts in Fungal Diversity and Community Structure Along the Altitudinal Gradient

The highest fungal richness values were found at sites M and S; however, the site M presented the highest Shannon index value (Table 4). On the other hand, the fungal community did not significantly (*p* > 0.05) vary in terms of evenness at the different altitudes and both Chao1 and ACE richness estimators showed that the site at the highest altitude (S) harbored the highest number of OTUs (Table 4). Mantel test and multiple-correlation analysis demonstrated that the only factor significantly (positively) correlating with the fungal diversity characteristics was soil pH (Table S1).

Fungal NMDS grouped the samples in four different clusters according to the sites they belonged to (Fig. 3b). According to PERMANOVA analysis, fungal communities at these sites significantly differed when all the samples were analyzed together (*p* < 0.001) or in pairwise comparisons (*p* < 0.05).

Mantel test demonstrated that the entire fungal community structure was positively and significantly correlated with all the environmental and chemical soil factors analyzed; i. e., the shifts in the fungal community over the altitudinal gradient are determined by the combination of several factors. Of all the variables examined, C/N, altitude, MAT, and MAST were most closely correlated with fungal community composition (Table 5).

The heat map analyzing the top 10 most abundant fungal classes (representing about 93 % of the classified and normalized sequences) showed that the relative abundance of these classes varied over the altitudinal gradient (Fig. 4b). Likewise, the UPGMA based on the relative abundance of these 10 classes grouped the sites according to the altitude; there were two clusters, one of them comprising the submontane and montane site (M and K) and another one grouping the subalpine and alpine sites (R and S; Fig. 4b). On the other hand, although the heat map graphically showed a change of the top 10 classes relative abundance with altitude, only the abundance of four (*Incertae sedis* 10 (*Zygomycota*), *Leotiomycetes*, *Eurotiomycetes*, and *Sordariomycetes* (*Ascomycota*)) of these 10 classes significantly varied between the four sites (Table S4). *Incertae sedis* 10 (including *Mucorales* and *Mortierales* orders) was more abundant at site R and was negatively correlated with C/N (Table S5). In the case of *Leotiomycetes*, a positive correlation between their relative abundance and altitude was found, being most abundant at site S. This fungal class also significantly correlated with most of the environmental and chemical factors analyzed (except pH and C/N; Table S5). *Eurotiomycetes* were present at a significantly higher abundance at site R, and any of the environmental factors tested significantly correlated with this group’s relative abundance. On the other hand, *Sordariomycetes* were most abundant at lower altitudes (site M), and unlike other fungal classes, this class significantly correlated with soil pH.

## Discussion

In the present study, archaeal, bacterial, and fungal communities were studied in quantitative and qualitative terms over the same altitudinal gradient. Regarding archaeal community, we did not detect shifts in the archaeal 16S rRNA gene copy number along the gradient; i.e., there was not a significant correlation between this group’s abundance and altitude, a finding that is consistent with the results described along other altitudinal gradient in Tibetan Plateau [1]. In our study, archaeal community only represented between 1.6 × 10^−4^ and 8.9 × 10^−4^ % of the prokaryotic community and the A/B ratio ranged from 1.60 × 10^−6^ to 8.91 × 10^−6^. Wang et al. [1] described that archaeal community accounted for 1–13 % of the total prokaryotic abundance in the aforementioned Tibetan gradient, and Bengtson et al. [44] assessed on average as 0.009 the ratio between archaeal and bacterial copy numbers in different arable soils. Therefore, archaeal abundance along the studied altitudinal gradient was much lower than that described in other surveys. The low archaeal abundance found in our study could be explained by the fact that archaea do not tend to be involved in litter decomposition in contrast to bacteria and fungi [45, 46], and we thus suppose that saprophytic microorganisms predominate in terms of abundance in the forest sites studied here. This extremely low archaeal abundance could explain the low amount of reads obtained for this domain in Illumina assay. The community composition analysis allowed us to conclude that the archaeal community was dominated by *Thaumarchaeota* phylum, which has previously been proven to dominate soil habitats [47]. The further classification of *Thaumarchaeotal* sequences as *Nitrososphaera* (genus) points to the dominance of ammonium oxidizers among the archaeal community at the studied sites, which concurs with other reports [48, 49].

Generally, it is expected to find a decrease (soil dry weight related) in bacterial and fungal abundances along mountain altitudinal gradients since a rise in altitude is related to an increase in environmental harshness [22]. However, we obtained a higher bacterial and fungal abundance at higher altitudes, which was significantly and positively related to the high amounts of C (TOC and humus), EC, N, and mineral nutrients in subalpine and alpine environments. Previous studies showed that also microbial activity at the studied sites increased with altitude [24]. The increased levels of C and other nutrients found at higher altitudes can be explained by the higher recalcitrance of coniferous litter, which produces a greater C sequestration [50] and lower nutrient immobilization rates [51]. The higher C, N, and P levels with altitude may contribute to enhance microbial growth, which could explain the higher microbial abundance at higher altitudes. The high SOM levels explain the increased EC values found at higher altitudes and their significant relation to microbial abundance, especially in the case of bacteria, since EC has been related to cation exchange capacity which increases with high levels of SOM [52].

Bacterial and fungal abundances along the altitudinal gradient were not influenced by soil pH. However, it was the most important factor determining the changes in bacterial and fungal diversities along altitude and also affected both community structures, especially those of bacteria. Soil pH does not alter microbial community itself but interacting directly or indirectly with other individual soil variables (e.g., enzymes activities, nutrient availability, organic C characteristics, salinity, or soil moisture) [53]. Since the pH range observed along the studied altitudinal gradient was 3.17–5.16, probably, these pH variations did not result in alterations of the soil characteristics governing changes in bacterial and fungal abundances; however, these pH variations did probably affect soil properties that are responsible for changes in bacterial and fungal structures as well as diversity. Previous studies have documented changes in soil bacterial community structure at very low pH scale (only 0.10) [54].

Although we found a negative correlation between altitude and bacterial richness/diversity along the altitudinal gradient, significant monotonic decreasing of both parameters along the altitudinal gradient were not observed, a finding that is in line with other studies which have suggested that bacteria may not follow the patterns of plants and animals along altitude [5, 10]. The significantly higher bacterial richness/diversity observed at the submontane site M could be related to the higher soil pH observed at this altitude, since higher bacterial diversity has been associated with higher soil pH values [55]. In our study, it is also worth noting that there were no significant differences in the structure of bacterial community between the K and S sites (according to NMDS and PERMANOVA analyses), although the dominant vegetation type and climatic conditions at both altitudes were completely different. Since the main factor governing bacterial community structure was soil pH according to Mantel test, the similar bacterial community structure at K and S sites could be attributed to soil pH, which was not significantly different at these two sites (4.10 at site M and 4.13 at site S). Generally, it is accepted that soil microbial communities’ structure over altitudinal gradients is mainly governed by changes in vegetation cover type [9]. However, the aforementioned result demonstrates that vegetation itself, except for specific microbes associated with certain trees, does not directly result in changes in soil microbial community structure but through indirect mechanisms which are also influenced by environmental factors [56].

The study of bacterial community composition along the studied sites was consistent with other surveys describing bacterial diversity over altitudinal gradients including deciduous and coniferous forests [9, 11]. A positive relation between Gram-negative bacteria and altitude as well as a negative relation between Gram-positive bacteria and altitude has been previously described [22]. This finding is in agreement with our results as *Gammaproteobacteria*, *Sphingobacteriia*, and *Opitutae* correlated positively with altitude, while *Actinobacteria* correlated negatively. The increase of the relative abundance of *Gammaproteobacteria* at higher altitudes, according to our data, is a consequence of the interaction of several climatic and soil chemical factors, such as MAT, MAST, MAP, pH, and nutrient and SOM contents. The negative correlation between *Gammaproteobacteria* and both mean annual air and soil temperature indicates an adaptation of these bacteria to cold conditions; e.g., soil warming experiments have shown a *Gammaproteobacteria* decrease after the incubation of soil at increasing temperature [57]. On the other hand, their positive relation with soil nutrient and SOM content could be a consequence of their copiotrophic lifestyle (r-strategists) [58]. On the other hand, the changes in the other proteobacterial classes were influenced by fewer environmental factors. *Betaproteobacteria*, regarded as r-strategists, related positively with SOM and nutrient contents, which is consistent with other works studying the ecological roles of these bacterial groups in soil [59, 60].

In the case of *Actinobacteria*, their decrease with altitude was related to changes in MAT, MAST, pH, and the higher concentration of SOM in subalpine and alpine sites (resulting in higher EC). These bacteria are adapted to resource-limited conditions and to life in the deep soil where competition between bacteria is less important [61].

*Acidobacteria* have been identified as one of the most common phyla in soil, with a dominance of the subgroups 1, 4, and 6. Especially abundant in soil is Gp1 [62], which was corroborated in our study. Soil pH has been described as one of the key factors influencing *Acidobacteria* community composition and structure in soil [63]. In fact, we found that Gp1 and Gp3 significantly correlated with soil pH. Many studies cataloging acidobacterial diversity in a high variety of soils have reported that most of the acidobacterial subgroups negatively correlate with C/N, which could be related to the adaptation of these bacteria to oligotrophic conditions [64], a finding that is in line with our findings for Gp2 and Gp3. Likewise, the negative correlation between C/N and Gp3 could be related to the participation of members of this group in the N cycle, according to a genomic study of cultivable Gp3 *Acidobacteria* [65].

According to our data, the fungal community did not exhibit any clear pattern in richness or diversity along the altitudinal gradient. Correlation analyses demonstrated that the only factor determining changes in fungal richness and diversity was soil pH. Similar conclusions were reached by Wang et al. [66] studying an altitudinal gradient in a Tibetan forest ecosystem. On the other hand, the structure of the fungal communities significantly differed along altitude in our study mainly because of changes in C/N according to Mantel test. However, soil pH was of minor significance for structuring fungal communities. Supporting these findings, Wang et al. [66] also reported, in the aforementioned gradient, that soil pH was not the dominant driver for fungal beta diversity. Likewise, Rousk et al. [55] concluded that the effect of pH was lower on fungal community composition than on bacterial community. Fungal community composition is often most closely associated with SOM-related characteristics, such as carbon and nutrient types as well as quality [67]. Our results support this fact since UPGMA based on the distribution of the most abundant fungal classes clustered the sites according to the vegetation zone.

The fungal community composition along altitude at phylum level (basically comprised of *Basidiomycota*, *Ascomycota*, and *Zygomycota*) is in line with that described for temperate forests at global scale [68]. In the present study, we found that, among the top 10 most abundant classes, the only one that showed a significant (and positive) correlation with altitude was *Leotiomycetes*. The shifts with altitude were negatively governed by air and soil temperature (MAT and MAST) and positively governed by precipitation (MAP) as well as the content of SOM and soil nutrients. Tedersoo et al. [68] also found a negative correlation between this fungal group and temperature at global scale, and the importance of precipitation on fungal dynamics has been previously highlighted [69]. *Leotiomycetes* encompasses diverse groups of ecologically different fungi, among them, specialized saprotrophs on wood and litter, a fact that could explain their positive relation with SOM content [70]. In the case of *Incertae sedis* 10 members, mainly *Mortierellales* and *Mucorales* (fast-growing soil-inhabiting saprotrophic fungi) orders in the present work, their changes over the sites were negatively correlated with C/N, which could indicate a special limitation of these fungi to grow with high C and low N levels. As fast-growing fungi, *Mortierellales* and *Mucorales* are adapted to degrade simple soluble substrates, such as pectin and easily accessible cellulose and hemicellulose [71], and need high amounts of N to keep their fast growing.

The results obtained in the present study allow us to conclude regarding our initial objectives that (i) archaeal abundance did not vary along the altitudinal gradient, while both bacterial and fungal community relative sizes increased with altitude since higher levels of SOM and nutrients were found at higher altitudes (first objective), and (ii) the composition of archaeal, bacterial, and fungal communities along an altitudinal gradient in European Alps was described for the first time. The diversity found for each community was consistent with that described for other forest soils and altitudinal gradients (second objective); (iii) although the submontane site showed higher bacterial and fungal diversity, it was not possible to detect any clear pattern in richness/diversity shifts of bacterial or fungal diversity along the altitudinal gradient (third objective), and (iv) the correlation analyses showed that environmental and soil chemical factors explain the variations in microbial communities’ properties better than altitude itself, and the different factors have a different effect on bacterial and fungal communities. The study of the variations of the main bacterial classes and their driving factors allowed us to prove that the demonstrated shifts are the result of a complex microbial interaction with the environmental factors prevailing at each site (fourth objective).

## Electronic Supplementary Material

The online version of this article contains supplementary material, which is available to authorized users.

ESM 1 **Table S1** Mantel test and multiple-correlation analysis results considering bacterial and fungal community diversity properties and the different environmental and chemical soil factors analyzed. Values in *bold* mean statistical significance. Significance level is shown at *p < 0.05 and **p < 0.01. **Table S2** Relative abundance of the top 15 most abundant bacterial classes found at M (545–570 m asl), K (1175–1200 m), R (1724–1737 m), and S (1965–2000 m) sites. For each class, data followed by *different letters* are significantly different according to Tukey’s HSD test (*p* ≤ 0.05). Significance level is shown at ^#^ *p* > 0.05; *p < 0.05, **p < 0.01. **Table S3** Multiple-correlation analysis results considering the top 15 most abundant bacterial classes and the different environmental and chemical soil factors analyzed. Values in *bold* mean statistical significance. Significance level is shown at *p < 0.05 and **p < 0.01. **Table S4** Relative abundance of the top 10 most abundant fungal classes found at M (545–570 m asl), K (1175–1200 m), R (1724–1737 m), and S (1965–2000 m) sites. For each class, data followed by *different letters* are significantly different according to Tukey’s HSD test (*p* ≤ 0.05). Significance level is shown at ^#^ *p* > 0.05; *p < 0.05, **p < 0.01. **Table S5** Correlation results (multiple-correlation analysis) considering the top 10 most abundant fungal classes and the different environmental and chemical soil factors analyzed. Values in *bold* mean statistical significance. Significance level is shown at *p < 0.05 and **p < 0.01. **Fig. S1** Bacterial rarefaction curves for M (M1–M4; 545–570 m asl), K (K1–K4; 1175–1200 m), R (R1–R4; 1724–1737 m), and S (S1–S4; 1965–2000 m) sites. **Fig. S2** Relative abundance of the different bacterial (**a**) and fungal (**b**) classes found at M (M1–M4; 545–570 m asl), K (K1–K4; 1175–1200 m), R (R1–R4; 1724–1737 m), and S (S1–S4; 1965–2000 m) sites. **Fig. S3** Fungal rarefaction curves for M (M1–M4; 545–570 m asl), K (K1–K4; 1175–1200 m), R (R1–R4; 1724–1737 m), and S (S1–S4; 1965–2000 m) sites. (PDF 538 kb)
