# Supplementary material for: Abundance and Diversity of Bacterial, Archaeal, and Fungal Communities Along an Altitudinal Gradient in Alpine Forest Soils: What Are the Driving Factors?
Source: Microb Ecol. 2016 Mar 9;72:207–20. doi: 10.1007/s00248-016-0748-2 (PMC4902835; doi:10.1007/s00248-016-0748-2)

SUPPLEMENTARY INFORMATION

Abundance and diversity of bacterial, archaeal and fungal communities along an altitudinal gradient in Alpine forest soils: what are the driving factors?

José A. Siles, Rosa Margesin

Corresponding author: **José A. Siles**

Email: Jose.Siles-Martos@uibk.ac.at

Microbial Ecology

The supplementary information includes Table S1, Table S2, Table S3, Table S4, Table S5 and Figure S1, Figure S2, Figure S3.

Table S1. Mantel test and multiple correlation analysis results considering bacterial and fungal community diversity properties and the different environmental and chemical soil factors analyzed. Values in bold mean statistical significance. Significance level is shown at: *, *p*<0.05 and **, *p*<0.01.

BACTERIAL COMMUNITY														
	**Overall**	**Altitude**	**MAT**	**MAST**	**MAP**	**pH**	**EC**	**Humus**	**TOC**	**N**	**C/N**	**P**	**K**	**Mg**
**Richness**	**0.505****	**-0.678****	**0.670****	**0.572***	**-0.583****	**0.862****	**-0.570***	**-0.603***	**-0.603***	**-0.593***	-0.437	-0.383	**-0.577***	-0.198
**Shannon**	**0.528****	**-0.727****	**0.721****	**0.635****	**-0.640****	**0.826****	**-0.676****	**-0.605***	**-0.605***	**-0.601***	-0.401	-0.443	**-0.544***	-0.193
**Evenness**	**0.481****	**-0.715****	**0.710****	**0.625****	**-0.639****	**0.740****	**-0.716****	**-0.600***	**-0.600***	**-0.588***	-0.397	-0.488	**-0.520****	-0.195
**Chao 1**	**0.558****	**-0.738****	**0.731****	**0.619***	**-0.655****	**0.821****	**-0.571***	**-0.616***	**-0.616***	**-0.606***	-0.451	-0.392	**-0.594***	-0.214
**ACE**	**0.647****	**-0.755****	**0.743****	**0.544***	**-0.681****	**0.604***	**-0.558***	**-0.536***	**-0.536***	-0.491	**-0.552***	-0.323	-0.489	-0.275
**FUNGAL COMMUNITY**														
	**Overall**	**Altitude**	**MAT**	**MAST**	**MAP**	**pH**	**EC**	**Humus**	**TOC**	**N**	**C/N**	**P**	**K**	**Mg**
**Richness**	0.021	-0.192	0.179	0.086	-0.038	**0.769****	-0.170	-0.089	-0.088	-0.042	-0.286	0.009	-0.162	0.351
**Shannon**	-0.058	-0.188	0.178	0.092	-0.076	**0.619***	-0.187	0.018	0.018	0.052	-0.173	0.039	-0.124	0.245
**Evenness**	-0.066	-0.190	0.181	0.098	-0.102	**0.519***	-0.200	0.032	0.032	0.060	-0.139	0.030	-0.122	0.166
**Chao 1**	-0.092	-0.052	0.044	0.036	0.079	**0.697****	-0.067	-0.061	-0.061	-0.028	-0.193	0.032	-0.102	0.376
**ACE**	-0.108	-0.087	0.080	0.072	0.033	**0.700****	-0.141	-0.172	-0.172	-0.141	-0.238	-0.080	-0.185	0.271

Table S2. Relative abundance of the top 15 most abundant bacterial classes found at M (545-570 m a.s.l.), K (1175-1200 m), R (1724-1737 m) and S (1965-2000 m) sites. For each class, data followed by different letters are significantly different according to Tukey's HSD test ($p \leq 0.05$). Significance level is shown at: #, $p > 0.05$; *, $p < 0.05$; **, $p < 0.01$.

**Class**	**Phylum**	**M**	**K**	**R**	**S**	***p*-value**
Alphaproteobacteria	Proteobacteria	6.40 **b**	7.04 **b**	4.82 **a**	6.74 **b**	**
Actinobacteria	Actinobacteria	7.86 **b**	7.23 **b**	3.72 **a**	6.19 **ab**	**
Gammaproteobacteria	Proteobacteria	3.73 **a**	6.71 **b**	7.54 **b**	7.02 **b**	**
**Gp1**	Acidobacteria	4.26 **a**	6.40 **ab**	9.08 **b**	5.26 **a**	**
Sphingobacteriia	Bacteroidetes	3.49 **a**	7.46 **b**	5.83 **ab**	6.02 **ab**	*
Betaproteobacteria	Proteobacteria	4.63 **a**	6.98 **a**	5.10 **a**	8.29 **b**	**
Planctomycetia	Planctomycetes	8.42 **b**	5.37 **ab**	6.68 **ab**	4.53 **a**	**
**Gp3**	Acidobacteria	6.75 **ab**	4.50 **a**	10.03 **b**	3.71 **a**	**
Deltaproteobacteria	Proteobacteria	7.22 **a**	7.27 **a**	4.69 **a**	5.83 **a**	#
**Gp2**	Acidobacteria	7.00 **ab**	2.69 **a**	11.79 **b**	3.53 **ab**	*
Spartobacteria	Verrucomicrobia	8.96 **a**	5.98 **a**	6.48 **a**	3.58 **a**	#
Subdivision3	Verrucomicrobia	3.69 **a**	6.59 **ab**	9.38 **b**	5.34 **a**	**
**Gp6**	Acidobacteria	10.83 **b**	4.15 **a**	3.85 **a**	6.18 **ab**	*
Clostridia	Firmicutes	4.71 **a**	0.36 **a**	5.55 **a**	14.39 **a**	#
Opitutae	Verrucomicrobia	3.44 **a**	6.87 **a**	7.26 **a**	7.43 **a**	#

Table S3. Multiple correlation analysis results considering the top 15 most abundant bacterial classes and the different environmental and chemical soil factors analyzed. Values in bold mean statistical significance. Significance level is shown at: *, p<0.05 and **, p<0.01.

Class	Phylum	Altitude	MAT	MAST	MAP	pH	EC	Humus	TOC	N	C/N	P	K	Mg
Alphaproteobacteria	Proteobacteria	-0.215	0.221	0.411	-0.134	**0.566***	-0.228	0.126	0.126	-0.056	0.482	0.017	0.157	0.485
Actinobacteria	Actinobacteria	**-0.542***	**0.544***	**0.613***	-0.466	**0.713***	**-0.586***	-0.146	-0.146	-0.274	0.212	-0.170	-0.114	0.210
Gammaproteobacteria	Proteobacteria	**0.694***	**-0.687***	**-0.563***	**0.628****	**-0.613***	**0.716****	**0.800****	**0.801****	**0.778****	**0.569****	**0.659****	**0.770****	**0.572***
**Gp1**	Acidobacteria	0.426	-0.420	-0.413	0.299	**-0.931****	0.342	0.374	0.374	0.400	0.226	0.139	0.313	-0.049
Sphingobacteriia	Bacteroidetes	**0.523***	**-0.522***	-0.338	**0.528***	-0.176	**0.676****	**0.849****	**0.849****	**0.751****	**0.704****	**0.831****	**0.899****	**0.830****
Betaproteobacteria	Proteobacteria	0.430	-0.425	-0.256	0.472	0.085	**0.636****	**0.717****	**0.717****	**0.638****	**0.567***	**0.720****	**0.708****	**0.862****
Planctomycetia	Planctomycetes	**-0.572***	**0.565***	0.373	**-0.578***	0.143	**-0.567***	**-0.745****	**-0.746****	**-0.669****	**-0.628****	**-0.654****	**-0.725****	**-0.659****
**Gp3**	Acidobacteria	-0.04	0.040	-0.142	-0.128	**-0.571***	-0.194	-0.351	-0.351	-0.193	**-0.520***	-0.473	-0.400	**-0.664****
Deltaproteobacteria	Proteobacteria	-0.439	0.443	**0.518***	-0.405	**0.574***	-0.451	-0.302	-0.302	-0.377	0.080	-0.259	-0.231	-0.023
**Gp2**	Acidobacteria	0.031	-0.038	-0.228	-0.029	-0.480	-0.108	-0.449	-0.448	-0.288	**-0.593***	**-0.507***	**-0.540***	**-0.722****
Spartobacteria	Verrucomicrobia	**-0.592***	**0.590***	0.465	**-0.611***	0.043	-0.457	**-0.510***	**-0.511***	**-0.519***	-0.408	-0.407	-0.438	**-0.539****
**Subdivision 3**	Verrucomicrobia	0.482	-0.476	-0.443	0.356	**-0.782****	0.345	0.261	0.261	0.350	0.089	0.121	0.304	-0.165
**Gp6**	Acidobacteria	-0.496	0.485	0.336	-0.408	**0.708****	-0.460	**-0.683****	**-0.683****	**-0.608***	**-0.612***	-0.469	**-0.722***	-0.367
Clostridia	Firmicutes	0.244	-0.250	-0.287	0.288	0.170	0.191	-0.230	-0.230	-0.151	-0.296	-0.056	-0.305	-0.224
Opitutae	Verrucomicrobia	**0.605***	**-0.598***	-0.472	**0.562***	-0.334	**0.562***	**0.548***	**0.548***	**0.564***	0.337	**0.551***	**0.643***	0.362

Table S4. Relative abundance of the top 10 most abundant fungal classes found at M (545-570 m a.s.l.), K (1175-1200 m), R (1724-1737 m) and S (1965-2000 m) sites. For each class, data followed by different letters are significantly different according to Tukey's HSD test ($p \leq 0.05$). Significance level is shown at: #, $p > 0.05$; *, $p < 0.05$; **, $p < 0.01$.

**Class**	**Phylum**	**M**	**K**	**R**	**S**	***p*-value**
Agaricomycetes	Basidiomycota	6.18 **a**	7.80 **a**	5.51 **a**	5.51 **a**	#
**Incertae sedis 10**	Zygomycota	7.55 **ab**	2.90 **a**	9.43 **b**	5.12 **ab**	*
Leotiomycetes	Ascomycota	3.41 **a**	4.37 **ab**	6.16 **ab**	11.06 **b**	*
Eurotiomycetes	Ascomycota	5.74 **ab**	7.66 **ab**	10.03 **b**	1.57 **a**	*
Dothideomycetes	Ascomycota	4.13 **a**	11.09 **a**	4.59 **a**	5.20 **a**	#
Tremellomycetes	Basidiomycota	8.15 **a**	4.72 **a**	1.55 **a**	10.58 **a**	#
Sordariomycetes	Ascomycota	13.13 **b**	1.87 **a**	2.83 **a**	7.18 **ab**	*
Archaeorhizomycetes	Ascomycota	5.37 **a**	16.02 **a**	2.56 **a**	1.06 **a**	#
Pezizomycetes	Ascomycota	1.92 **a**	2.64 **a**	8.06 **a**	12.38 **a**	#
Microbotryomycetes	Basidiomycota	3.64 **a**	6.27 **a**	9.91 **a**	5.19 **a**	#

Table S5. Correlation results (multiple correlation analysis) considering the top 10 most abundant fungal classes and the different environmental and chemical soil factors analyzed. Values in bold mean statistical significance. Significance level is shown at: *, *p*<0.05 and **, *p*<0.01.

Class	Phylum	Altitude	MAT	MAST	MAP	pH	EC	Humus	TOC	N	C/N	P	K	Mg
Agaricomycetes	Basidiomycota	-0.188	0.196	0.308	-0.209	0.074	-0.185	-0.260	-0.260	-0.414	0.253	-0.188	-0.167	-0.207
**Incertae sedis 10**	Zygomycota	-0.001	-0.011	-0.251	-0.025	-0.273	0.015	-0.161	-0.160	0.083	**-0.642****	-0.144	-0.188	-0.297
Leotiomycetes	Ascomycota	**0.620***	**-0.625****	**-0.602***	**0.673****	-0.052	**0.721****	**0.746****	**0.746****	**0.789****	0.259	**0.708****	**0.599****	**0.744****
Eurotiomycetes	Ascomycota	-0.143	0.149	0.133	-0.257	-0.448	-0.212	-0.320	-0.320	-0.265	-0.204	-0.408	-0.254	-0.492
Dothideomycetes	Ascomycota	-0.039	0.053	0.255	-0.078	0.123	0.026	0.291	0.291	0.136	**0.520***	0.267	0.267	0.244
Tremellomycetes	Basidiomycota	-0.023	0.017	0.012	0.089	0.470	-0.195	-0.084	-0.084	-0.072	-0.183	-0.070	-0.047	0.147
Sordariomycetes	Ascomycota	-0.424	0.411	0.234	-0.323	**0.513***	-0.319	-0.280	-0.280	-0.235	-0.453	-0.162	-0.269	-0.129
Archaeorhizomycetes	Ascomycota	-0.266	0.279	0.444	-0.308	-0.056	-0.235	0.096	0.096	-0.090	**0.570***	-0.003	0.203	0.0847
Pezizomycetes	Ascomycota	0.465	-0.469	-0.481	0.487	-0.241	0.072	0.092	0.092	0.176	-0.071	-0.089	0.027	0.0419
Microbotryomycetes	Basidiomycota	0.281	-0.278	-0.277	0.206	-0.291	0.057	-0.186	-0.187	-0.054	-0.273	-0.199	-0.197	-0.402

Fig. S1. Bacterial rarefaction curves for M (M1-M4) (545-570 m a.s.l.), K (K1-K4) (1175-1200 m), R (R1-R4) (1724-1737 m) and S (S1-S4) (1965-2000 m) sites.

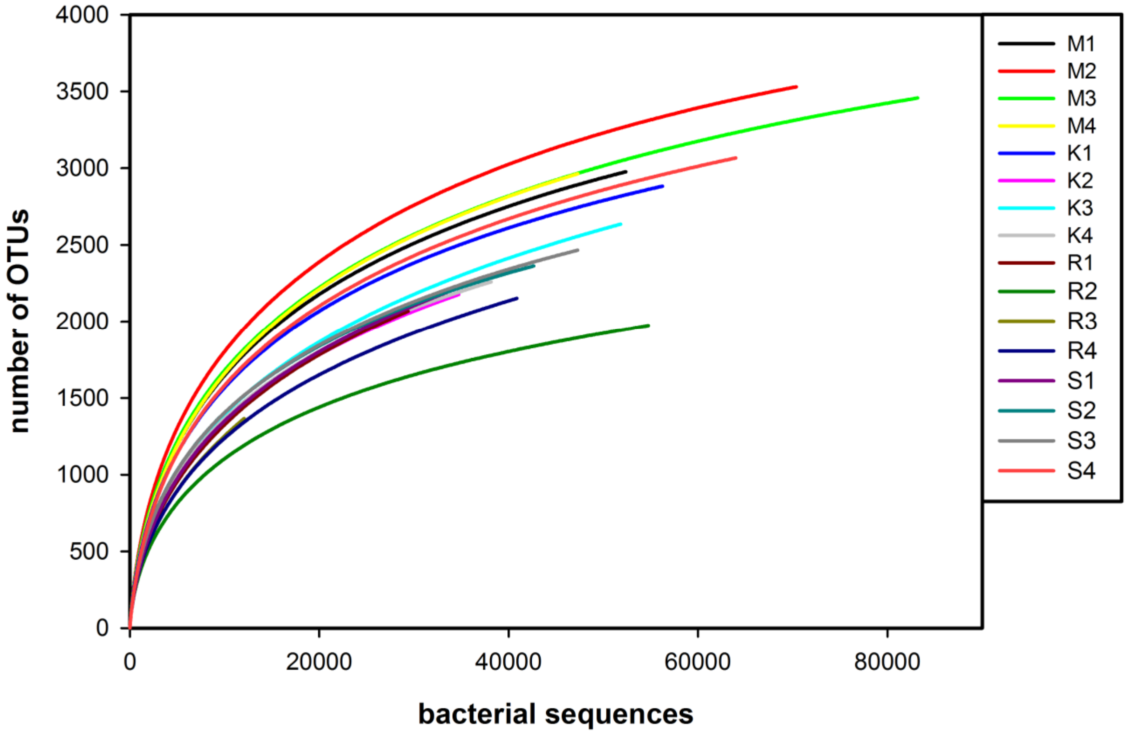

Fig. S2. Relative abundance of the different bacterial (a) and fungal (b) classes found at M (M1-M4) (545-570 m a.s.l.), K (K1-K4) (1175-1200 m), R (R1-R4) (1724-1737 m) and S (S1-S4) (1965-2000 m) sites.

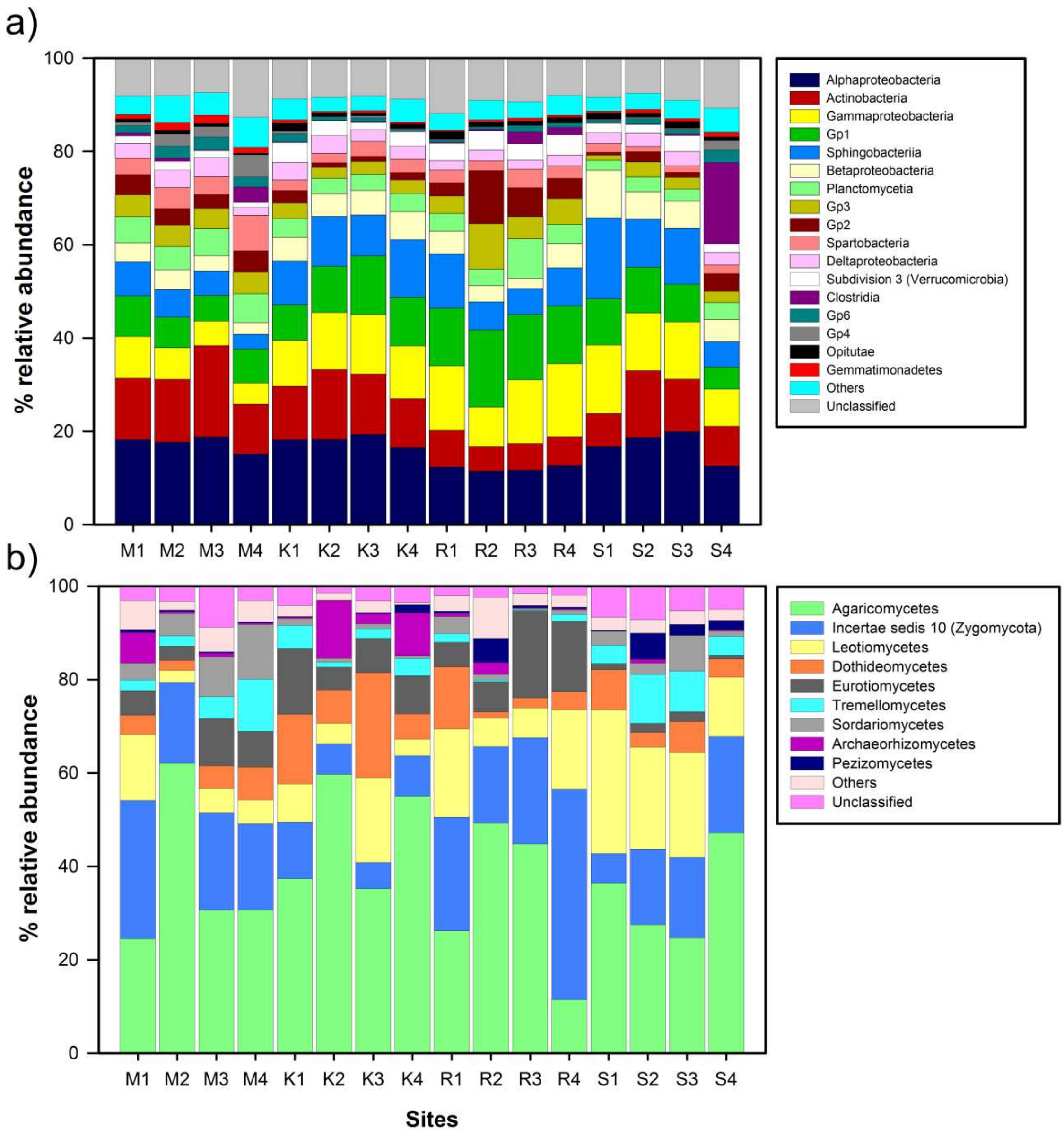

Fig. S3. Fungal rarefaction curves for M (M1-M4) (545-570 m a.s.l.), K (K1-K4) (1175-1200 m). R (R1-R4) (1724-1737 m) and S (S1-S4) (1965-2000 m) sites.

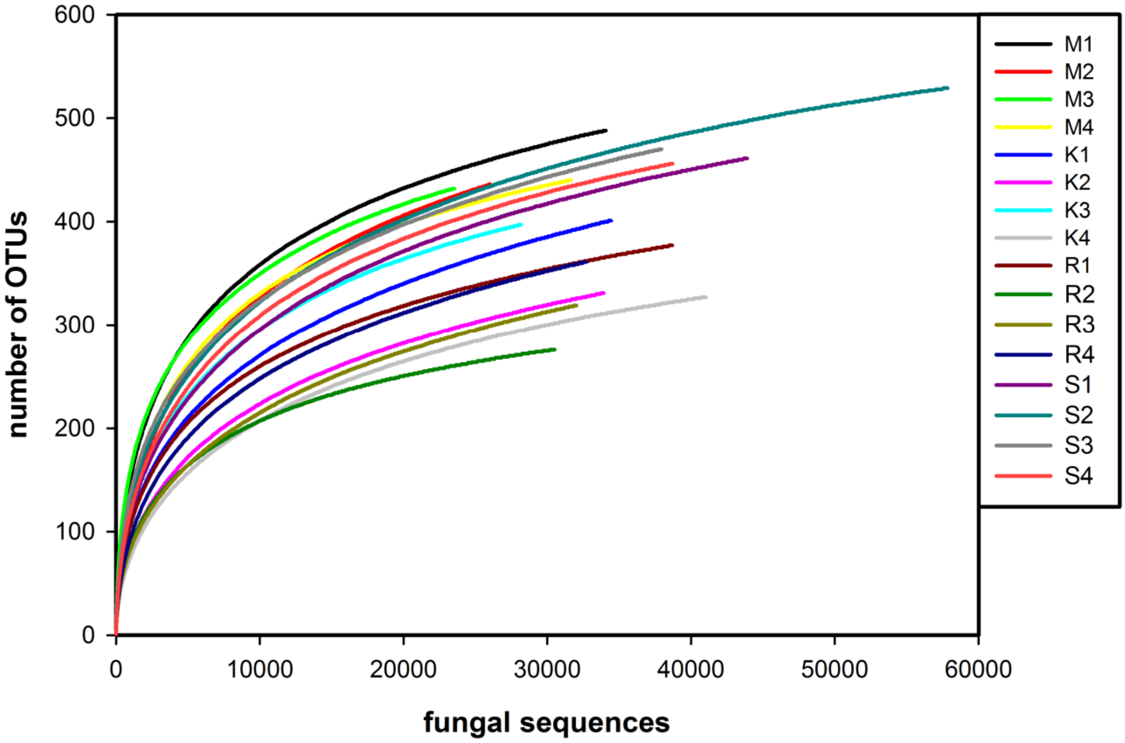

Supplement: Supplementary file 1 — Table S1 Mantel test and multiple-correlation analysis results considering bacterial and fungal community diversity properties and the different environmental and chemical soil factors analyzed. Values in bold mean statistical significance. Significance level is shown at *p < 0.05 and **p < 0.01. Table S2 Relative abundance of the top 15 most abundant bacterial classes found at M (545–570 m asl), K (1175–1200 m), R (1724–1737 m), and S (1965–2000 m) sites. For each class, data followed by different letters are significantly different according to Tukey’s HSD test (p ≤ 0.05). Significance level is shown at # p > 0.05; *p < 0.05, **p < 0.01. Table S3 Multiple-correlation analysis results considering the top 15 most abundant bacterial classes and the different environmental and chemical soil factors analyzed. Values in bold mean statistical significance. Significance level is shown at *p < 0.05 and **p < 0.01. Table S4 Relative abundance of the top 10 most abundant fungal classes found at M (545–570 m asl), K (1175–1200 m), R (1724–1737 m), and S (1965–2000 m) sites. For each class, data followed by different letters are significantly different according to Tukey’s HSD test (p ≤ 0.05). Significance level is shown at # p > 0.05; *p < 0.05, **p < 0.01. Table S5 Correlation results (multiple-correlation analysis) considering the top 10 most abundant fungal classes and the different environmental and chemical soil factors analyzed. Values in bold mean statistical significance. Significance level is shown at *p < 0.05 and **p < 0.01. Fig. S1 Bacterial rarefaction curves for M (M1–M4; 545–570 m asl), K (K1–K4; 1175–1200 m), R (R1–R4; 1724–1737 m), and S (S1–S4; 1965–2000 m) sites. Fig. S2 Relative abundance of the different bacterial (a) and fungal (b) classes found at M (M1–M4; 545–570 m asl), K (K1–K4; 1175–1200 m), R (R1–R4; 1724–1737 m), and S (S1–S4; 1965–2000 m) sites. Fig. S3 Fungal rarefaction curves for M (M1–M4; 545–570 m asl), K (K1–K4; 1175–1200 m), R (R1–R4; 172 [file 248_2016_748_MOESM1_ESM.pdf]
